# The Effects of COVID-19 Pandemic and Lockdown on Pediatric Nutritional and Metabolic Diseases: A Narrative Review

**DOI:** 10.3390/nu15010088

**Published:** 2022-12-24

**Authors:** Maria Elena Capra, Brigida Stanyevic, Antonella Giudice, Delia Monopoli, Nicola Mattia Decarolis, Susanna Esposito, Giacomo Biasucci

**Affiliations:** 1 Pediatrics and Neonatology Unit, Guglielmo da Saliceto Hospital, 29121 Piacenza, Italy; 2 Pediatric Clinic, Department of Medicine and Surgery, University of Parma, 43126 Parma, Italy

**Keywords:** COVID-19, eating disorders, lockdown, type 1 diabetes mellitus, type 2 diabetes, obesity

## Abstract

SARS-CoV-2 was the first pathogen implied in a worldwide health emergency in the last decade. Containment measures have been adopted by various countries to try to stop infection spread. Children and adolescents have been less clinically involved by COVID-19, but the pandemic and consequent containment measures have had an important influence on the developmental ages. The COVID-19 pandemic and the subsequent lockdown periods have influenced the nutrition and lifestyles of children and adolescents, playing an epigenetic role in the development of nutrition and metabolic diseases in this delicate age group. The aim of our review is to investigate the effects of the COVID-19 pandemic on nutrition and metabolic diseases in the developmental ages. Moreover, we have analyzed the effect of different containment measures in children and adolescents. An increase in being overweight, obesity and type 2 diabetes mellitus has been detected. Concerning type 1 diabetes mellitus, although a validated mechanism possibly linking COVID-19 with new onset type 1 diabetes mellitus has not been yet demonstrated, barriers to the accessibility to healthcare services led to delayed diagnosis and more severe presentation of this disease. Further studies are needed to better investigate these relationships and to establish strategies to contain the nutritional and metabolic impact of new pandemics in the developmental ages.

## 1. Introduction

COVID-19 has been the cause of high mortality and morbidity rates all over the world [1], causing millions of infections and deaths per day since April 2020 [2]. Between the start of the pandemic and 5 October 2022, there were 616,427,419 confirmed cases of COVID-19, including 6,528,557 deaths. Since the 4 October 2022, a total of 12,723,216,322 vaccine doses have been administered [3]. SARS-CoV-2 was the first pathogen implied in a worldwide health emergency during the last decade. However, SARS-CoV-2 infection is rarely complicated by life-threatening conditions in children, and the disease is often asymptomatic or pauci-symptomatic [1,4].

At the beginning of the pandemic, various strategies have been adopted to try to control and prevent the spread of the infection, with an urge for a rapid availability and development of effective treatments and vaccines [5]. In general, several strategies can be implemented to minimize the transmission rate of a particular epidemic. In the case of COVID-19, social distancing has been adopted by several countries as the main measure to contain the spread of the pandemic. Isolation and quarantine have been two important measures to stop further spread of the disease. Isolation was applied to already infected individuals, whereas quarantine was generally applied in cases of apparently healthy, but probably infected subjects, due to strict contact with SARS CoV2-infected individuals. In each country, according to the local health authorities recommendations, and the socio-economic and political status, different models of quarantine were applied, with different consequences on the everyday life and activities of the whole population, including children [6].

In a recent metanalysis, the COVID-19 lockdown resulted in a worse clinical control in adult patients with type 2 diabetes mellitus, with a significant increase in the levels of glycated hemoglobin (HbA1C), fasting glucose and body mass index [7]. COVID-19 has also been linked, in the adult population, with an increased risk of developing diabetes, due to interactions with the angiotensin-converting-enzyme 2 receptors [8]. 

In this context, the COVID-19 pandemic and the subsequent lockdown periods have influenced nutrition and lifestyle in children and adolescents, therefore playing an epigenetic role in the development of nutrition-related and metabolic diseases in this sensitive age. Due to their dynamic nature, epigenetic mechanisms are influenced by the environment, and the epigenome has the function of an interface between the environment and the genome. Pollutants, nutritional and hormonal factors, and drugs can have a positive or negative effect on epigenetic mechanisms, and this effect can vary depending on sex and tissue [7,8,9,10]. An epigenetic memory of multiple environmental conditions (e.g., diet, air pollution, noise pollution, lifestyle, work, family, education and physical activities) acquired during early life, is a long-lasting factor that can influence adult phenotype as well as being epigenetically inherited from generation to generation [11,12,13,14,15]. An unhealthy diet, high in lipids and too low or too high in protein intake, can address epigenetic responses associated with metabolic diseases, e.g., cardiovascular diseases (CVD), type 2 Diabetes Mellitus (T2DM), obesity and hypertension, that will become evident and last up to adulthood [16].

The aim of our review is to investigate the relationship between the COVID-19 pandemic on nutrition and metabolic diseases in the developmental ages. Moreover, we have also analyzed the effect of containment measures on nutritional and metabolic issues in children and adolescents. We have focused on these two aspects, as we think they may be more likely to be influenced by infectious diseases and environmental alterations. In-depth research and review of the medical literature was performed. The MEDLINE–PubMed database was searched from 2014 to 2022 to collect the literature. The search included randomized placebo-controlled trials, controlled clinical trials, double-blind, randomized controlled studies and systematic reviews of the last five years. The following combinations of keywords were used: “COVID-19” AND “nutrition” OR “overweight” OR “obesity” OR “eating disorders” OR “anorexia” OR “bulimia” OR “type 1 diabetes” OR “type 2 diabetes” AND “children” OR “paediatric” OR “pediatric” OR “adolescent”. We also performed the manual search of the reference lists of the obtained studies. The search was limited to English-language journals and full papers only. 

## 2. The Psychological Impact of Lockdown in Childhood and Adolescence

After the declaration of the pandemic, lockdown was initiated in various countries and aimed at limiting people’s movements and protecting national borders from foreign agents that could further spread the disease [17,18]. Everyday life was dramatically transformed for children and adolescents. As a consequence of the lockdown, psychological effects such as anxiety, depression, irritability, mood swings, inattention and sleep disturbance became fairly common not only among quarantined, but also among isolated children [19]. 

The psychosocial impact of social isolation in childhood and adolescence should not be underestimated, especially in the case of the prolonged duration of the lockdown, as children and adolescents may develop fears of infection, frustration, boredom and endure the negative effects of discontinued direct contact with classmates, friends and teachers, lack of privacy at home, intra-familial conflicts and the adverse effects of economic loss [20]. Different types of lockdowns put into action in different countries all over the world [21,22,23,24,25,26] during the COVID-19 pandemic are summarized in Table 1. In Turkey, an age-stratified curfew, involving youth but not adults, was applied. This containment measure was often perceived as a suppression of personal will, plans and decisions, with subsequent psychological consequences [24]. Italian surveys [27] highlighted that the COVID-19 pandemic had serious psychological consequences in children and adolescents, causing additional stress and acting as a potential trigger for the onset of neuropsychological disorders. Suicidal ideation, depression and eating disorders were the most frequently reported neuropsychological disorders. 

## 3. Eating Disorders

Eating disorders (EDs) represent relevant mental health diseases often associated with impairments in physical health and social, emotional and cognitive development. The onset of EDs in childhood and/or in adolescence can influence identity formation and self-esteem [28]. According to the Diagnostic and Statistical Manual of Mental Disorders Fifth Edition (DSM-5), the main EDs are anorexia nervosa (AN), bulimia nervosa (BN) and binge eating disorder (BED) [29].

EDs mostly affect girls during adolescence or late childhood, although in recent years the diagnosis of EDs has also become more frequent in children under 10 years of age [30]. From 1999 to 2002, in the United States, there was a 72% increase of hospitalizations for EDs in the age group 12–18 years [31]. The peak age of onset for anorexia nervosa (AN) and bulimia nervosa (BN) is mid-adolescence and late adolescence, respectively. Even if cumulative incidence rates have not changed in the last several years, there has been an important increase in the incidence of AN in 15- to 19-year-old girls [32]. The lifetime prevalence of AN, BN and BED in adolescent females is 0.3%, 0.9% and 1.6%, respectively [33]. A systematic literature review, including 94 studies, showed that the lifetime overall prevalence of EDs is 8.4% in females and 2.2% in males. As for single diseases, the lifetime prevalence of AN and BN is 1.4% in females and 0.2% in males, and 1.9% in females and 0.6% in males, respectively, whereas BD affects 2.8% of females and 1.0% of males [34]. Although the reported female-to-male ratio is 9:1, an increasing number of males with EDs is being diagnosed, especially among younger subjects [35].

Although the first case of an adolescent patient with an ED has been described more than 300 years ago, the pathophysiology and psychobiology of EDs have not yet been fully clarified [36]. Certainly, EDs have multiple biological and psychosocial risk factors, including genetic and environmental factors [37] such as: elevated weight, shape concerns, negative self-evaluation, sexual abuse and other adverse experiences [38]. Problematic eating behaviors are described in the general population and individuals with pre-existing eating disorders might be particularly vulnerable [39].

An increased incidence, prevalence and/or acuity of EDs during the COVID-19 pandemic have been reported. In a tertiary care children’s hospital in Boston, dealing with ED patients aged 8 to 26 years, an increase in the monthly admission rate was observed (slope = 1.22, *p* = 0.006) during the pandemic, compared to the pre-pandemic period when the number of admissions remained constantly stable (slope = −0.03, *p* = 0.78) [40,41]. In Italy, the Italian Society of Pediatrics described an increase in ED diagnoses in Italian children (+78.4%) as well as an increase in the need for admission to pediatric ED centers (+31.4%), due to restrictions in everyday activities, stress and exposure to social media. In a retrospective multicenter observational study carried out by the Italian Society of Pediatrics, a 78.4% increase of ED admissions of children into pediatric emergency departments was described from March 2020 to March 2021. Interference with everyday activities and routines, stress and increased exposure to social media have been identified as the predictors of the onset of EDs in children and adolescents during the first wave of COVID 19 [27].

Although social media and smartphones have been crucial for children and adolescents for communication during the restraints, their pathological use has been widely linked with promoting self-injurious behaviors and EDs. Platforms such as “Tik Tok” have recently been blamed for the spread of pro-anorexia, pro-suicide and self-harm videos [41]. In addition, the socio-economic gap must be considered, in order to understand how the lockdown impacted eating behaviors and EDs in the pediatric population. In a study performed in Brazil, social isolation was proven to affect and worsen the eating habits of children and adolescents, especially those belonging to lower-class families. Children coming from low-income families often experienced inadequate housing conditions, aggravated by the lack of access to drinking water, basic sanitation and healthy food, and they had many meals based on sweets, soft drinks and packaged salty snacks [42].

The lockdowns during the COVID-19 pandemic have also changed the regular daily routine. In the United Kingdom, Dawn Branley-Bell et al. described the effect of lockdowns on patients with EDs aged 16–65 years. The majority of them (86.7%), reported an exacerbation of their symptoms during the pandemic, also as a result of changes in their approach to food, and of the loss of a regular routine that contributed to the individual’s sense of control [43]. Unfortunately, data reported in the study were not subdivided and analyzed for the different age groups. Although the pandemic has represented a risk factor for the development of EDs, it has also influenced and allowed the spread of new remote follow-up strategies, such as telemedicine. A study carried out in Israel analyzed the ED treatment at the Sheba Medical Center in the Safra Children’s Hospital in the first 10 months of 2020 and compared it to the same period of months in the previous five years (2014–2019). Despite a slightly lower number of patients being treated in 2020 (242 patients, 127 new patients in 2020 vs. 257 patients, a mean of 166 new patients per year from 2014–2019), the use of telemedicine made it possible to overcome many obstacles. Indeed, the use of telemedicine can increase access through a reduction of limitations such as travel time, competing responsibilities and/or absence from work, meanwhile providing some advantage for health care professionals and institutions, including schedule flexibility, increased productivity and fewer clinic overheads [44]. Indeed, an increase of telemedicine sessions has been observed, most likely because telemedicine had not been utilized prior to the COVID-19 out-break, and secondly due to the social restrictions adopted during the first lockdown in Israel (all people had to stay at home). Furthermore, lockdown may have increased tension and distress and, in turn, also ED-related symptoms, collectively requiring more intensive treatment. 

Positive outcomes during the COVID-19 lockdown for adolescents with EDs have been presented by Akgul S et al., highlighting the importance of family-based therapy. This study was conducted in Turkey, where a unique age-stratified lockdown was implemented for children and youth under the age of 20 years during the period 21 March–11 June 2020 [24,45]. The family-based approach was aimed at empowering families in their effort to assist their children, and required parents to take full charge of the disorder during the acute phase. This may be easier when the child/adolescent and parents are all together in a closed and controlled environment as it had occurred during the pandemic [46].

In conclusion, the lockdown had both negative and positive outcomes in relation to EDs. Although isolation increased mental health disorders including EDs, it has been an opportunity to develop new approaches of medical treatment, such as telemedicine and family-based therapy. The different lockdown strategies adopted in various countries and the available data on nutritional diseases are summarized in Table 2.

## 4. Obesity and Being Overweight 

Obesity is a multifactorial disease involving a complex interaction between genetic, environmental and behavioral factors [48]. According to the World Obesity Federation, childhood obesity is increasing worldwide. In 2019, it was estimated that 158 million children aged 5 to 19 years were affected by obesity. Most of these children live in low-to-middle-income countries, and this number is projected to increase to 254 million by 2030 [49]. In Western countries, approximately 20% of children are obese [50,51]; the onset of obesity is often in preschool years and the risk to a child of remaining obese increases through adolescence into adulthood [52,53]. The long-term consequences of obesity include high blood pressure, Type 2 Diabetes Mellitus (T2DM), metabolic syndromes and cardiovascular diseases [54,55]. The COVID-19 pandemic contributed to the increase in childhood obesity by affecting environmental and behavioral factors. 

The ‘stay-at-home’ message has been one of the strategies adopted worldwide to try to slow down the spread of COVID-19, but this policy may have had a negative effect on obesity treatment [56]. Pandemic restrictions such as social isolation, school closures and the loss of regular routines, acted as stressors and disruptors in children’s lives [57,58,59]. These restrictions have led to significant changes in the lifestyles of the pediatric population, which include changes in sleep patterns and eating behavior, increased anxiety and fear, increased media consumption, decreased sports activity and, overall, more sedentary behaviors [60,61,62,63,64]. Pre-COVID-19 studies reported that obesogenic behaviors, such as a sedentary lifestyle, a poor diet, increased screen time and irregular sleep, are beneficially regulated when children follow a structured daily routine [65]. During the pandemic “stay-at-home”, parents were often on “smart working” schedules, or worried by the pandemic situation; therefore, it was often difficult to establish and maintain a definite daily routine for children and adolescents. Rundle et al. found that children living in urban areas or within small apartments were usually dealing with limited space or opportunities for physical activity and were consequently more susceptible to weight gain [57]. In addition, overweight and/or obese children who followed lifestyle weight-control programs had worse results at home than when they were engaged in normal daily activities, such as school and sports. Indeed, this demonstrates that this population was significantly and negatively affected by the lockdown. 

According to von Hippel et al., the obesity rate increased during the summer vacation by an average of 0.85 percentage points per month (95% confidence intervals [C.I.] 0.58–1.12), but decreased during each school year [66]. Franckle et al. ascribed this trend to the reduced physical activity during the summer holidays [67]. Lockdown and isolation periods may be considered as an “early onset summer holiday” [68] and, therefore, weight-gain during lockdown may be due to a similar cause [69].

Some studies have also shown the worrying association between decreased physical activity and increased food consumption of snacks and high-caloric food [70,71,72], through direct and deliberate buying in supermarkets [57]. An observational study conducted in Italy found a substantial increase in consumption of high-caloric food during the lockdown because of so called “stress-eating” [73]. Authors also reported an increase in the time spent on a screen of up to about five hours per day [73]. This effect is particularly worrisome as Fang et al. found that a screen time of ≥2 h per day significantly increased the risk of weight gain among children compared to a screen time of <2 h per day [74]. Despite a higher screen time having been of some benefit for educational aims and social communication among children, this can further promote sedentary habits and increase the risk of obesity [75].

In conclusion, several studies have shown that during the COVID-19 pandemic children, adolescents and young adults have gained excessive weight with respect to their estimated weight gain for age [76,77,78,79,80,81,82]. In this context, the need for an integrated approach to promote physical activity, lifestyle counseling and psychological support seems to be crucial [83,84,85,86]. Moreover, obesity is a risk factor for COVID-19 infection and infected patients with obesity are more likely to experience a severe form of the disease [87,88,89,90,91]. Previous research has shown that a high body mass index (BMI) was correlated with an increased need for mechanical ventilation in adult patients with a BMI > 35 kg/m2 [92], and this correlation was independent of the presence of any other comorbidities, gender or age [93,94,95,96,97]. Therefore, it seems reasonable to recommend the COVID-19 vaccination to obese patients, especially those with a higher BMI [98].

## 5. Type 1 Diabetes Mellitus

Type 1 diabetes mellitus (T1DM) is an autoimmune disorder with a permissive genetic background influenced by various environmental factors, such as junk food or viral infections [99]. These environmental factors act as an autoimmune stimulation and eventually lead to the partial or complete destruction of pancreatic beta-cells, causing insulin deficiency. T1DM constitutes about 5% of all diagnosed cases of diabetes and its global incidence is increasing by approximately 3% every year [100]. Various viruses, such as enteroviruses, coxsackie B, coxsackie A, cytomegalovirus, rotaviruses and retroviruses, have been linked to the pathogenesis of T1DM [101,102]. Pre-existing diabetes mellitus is supposed to be one of the high-risk factors for developing COVID-19 and related complications [103]. Indeed, there are many reports of COVID-19-induced severe metabolic alteration of pre-existing or new-onset diabetes such as diabetic ketoacidosis (DKA) and hyperglycemic hyperosmolar state (HHS) [104,105].

An increased number of children with newly diagnosed T1DM has been reported during the COVID-19 pandemic [106]. Several reports from countries heavily impacted by the pandemic suggested that more children with new-onset T1DM presented with severe decompensation, which is one of the most frequent and life threatening acute complications of T1DM [107]. In addition, psychiatric disorders, stress, lower socioeconomic status and high HbA1C values are linked to an increased risk of DKA [108]. Furthermore, infections are one of the main predisposing factors for DKA in diabetic patients, as well as insufficient insulin therapy [109].

Because of a paucity of epidemiological studies, it is still unclear whether there has been a true COVID-19-related increase in the T1DM incidence rather than just an exacerbation of the disease presentation with metabolic complications, such as DKA. SARS-CoV-2 is able to mediate islet cell damage, as already evidenced by previous coronaviruses epidemics [110]. Islet cell damage may occur without an activation of the immune system. [111]. In fact, in a small study conducted in the UK among children with newly diagnosed T1DM, SARS-CoV-2 was detected by polymerase chain reaction (PCR) in 2/21 and SARS CoV-2 antibodies in 3/16 children tested. COVID-19 infection cannot be ruled out only by a negative PCR test for SARS-CoV-2; indeed, computed tomography-positive cases of COVID-19 infection in patients with SARS-CoV-2 RNA-negative tests have been described [112]. Regional differences in prevalence and T1DM incidence in migrants conforming to the incidence in the country of destination, and the north-south gradient with higher figures in northern latitudes, have supported the role of environmental influences. In addition to this, the well-established seasonality of new-onset T1DM has also led to the investigation of possible viral etiology [113].

Indeed, some studies from high-income countries (Italy, Germany, UK, Australia and Finland) reported a higher incidence of DKA in pediatric patients with newly diagnosed T1DM during the COVID-19 pandemic, suggesting that there may be a correlation between infection and the onset of T1DM or DKA [114,115,116,117]. Conversely, a study performed in Alberta, Canada, demonstrated that the incidence of T1DM did not differ significantly during the COVID-19 pandemic, whereas a significant increase in the incidence of DKA was reported in the same period [118]. This suggests that a delay in the diagnosis of children with the new onset of T1DM, is likely due to the fear of referring to hospital, and to the under-recognition of symptoms due to the reduced direct interaction with health care professionals. However, all these studies emphasized the importance of training health care professionals and families to be aware of the symptoms of hyperglycemia and stressed early diagnosis and treatment of T1DM, also during a pandemic or a disaster, as issues of the utmost importance. 

Two studies conducted in Germany [104] and in Italy [116] reported no increase in the incidence of T1DM in children during the COVID-19 pandemic and attributed this finding to a reduced exposure to seasonal viruses. During the lockdowns, children did not attend school, and this led to a reduction in interpersonal interactions and reduced the chance of coming into contact with seasonal pathogens, which are a recognized precipitating factor for T1DM. Also, these studies did not report an increase in acute complications of diabetes during the first 40 days of the pandemic, with fewer DKA and severe hypoglycemia cases compared to the same period of the previous year. 

In another study, Tornese et al. [119] reported that adolescents with TDM1 using hybrid closed-loop systems did not have a worse glycemic control during the COVID-19 restrictions. Moreover, the glycemic control was better if physical activity was not discontinued due to containment measures. A possible explanation is that during quarantine the fear of becoming ill and the presence of parents/guardians at home may have improved self-care and have increased the supervision of diabetes control. 

It has been hypothesized that SARS-CoV-2 can be an environmental trigger for diabetes in individuals with high genetic risk and pre-existing beta-cell autoimmunity. It has been also reported that it may have a direct cytotoxic effect on beta-cells trough the bond of the angiotensin converting enzyme 2 (ACE2) receptor or by the proteolytic cleavage of the viral spike protein by the serine transmembrane protease 2 (TMPRSS2) [120,121]. ACE2 typically degrades Angiotensin II to Angiotensin [23,24], one of the active heptapeptides in the renin–angiotensin system. Angiotensin II can cause a reduction in insulin secretion and in blood flow to islet cells, with the consequent onset of hyperglycemia, local inflammation, apoptosis and decreased proliferation of islet cells [122]. Angiotensin counteracts this effect by increasing insulin secretion and vasodilation. SARS-CoV-2 binding to ACE2 may result in the downregulation of ACE2, leading to the unopposed action of Angiotensin II. In addition to this, a follow-up study, investigating pathogenesis of pancreatic lesions, also found that pancreatic islets are strongly immune-positive for ACE2, while exocrine tissues are only weakly positive [110]. 

In summary, in spite of the different outcomes in the development of new-onset T1DM, these studies have reported a significant increase in DKA and severe DKA in the diagnosis of diabetes in childhood and adolescence during the COVID-19 pandemic [115,123].

Alfayez el al. published a systematic review and meta-analysis focusing on the risk of DKA and severe DKA during the COVID-19 pandemic versus the prior-to-COVID-19 period among pediatric patients with T1DM and showed that the risk of DKA and severe DKA significantly increased during the pandemic [124]. Given the accepted theory of the pathogenesis of T1DM and the worldwide increase in the incidence of severe T1DM, DKA and severe DKA in children during the recent pandemic, it can be hypothesized that SARS-CoV-2 is likely to be a trigger for the autoimmune system, especially for pancreatic autoimmunity and the initiation of T1DM. More high quality, long-term studies are needed to better clarify this effect. On the other hand, if SARS-CoV-2 induces the onset of T1DM and/or its complications, T1DM may be associated with increased susceptibility to the viral infection. Indeed, hyperglycemia, by altering the immune response and causing cytokine dysregulation, represents, per se, a proinflammatory and procoagulant condition [125,126].

In conclusion, a validated mechanism that connects SARS-CoV-2 infection with new onset T1DM has not been yet demonstrated; nevertheless, changes in the availability of healthcare services and parental fears about accessing hospitals due to the SARS-CoV2 epidemic may have created barriers to the accessibility of healthcare services, leading to delayed diagnosis and more severe presentation of T1DM. Information campaigns on T1DM prevention and early diagnosis resulted in effectively reducing these potentially life-threatening acute complications also before the COVID-19 pandemic [127]. These campaigns might prove to be effective even when health systems are under pressure. 

## 6. Type 2 Diabetes Mellitus

T2DM is caused by a combination of inadequate insulin secretion combined with reduced insulin sensitivity [128,129]. It is well known that youth with T2DM often develop related complications within a few years from diagnosis [128,130,131]. Given its incidence increase, T2DM is considered a public health challenge in the pediatric population [132,133]. 

The role of SARS-CoV2 and the lockdowns in the incidence and development of this disease during the pandemic has been described by several studies in literature. In a cross-sectional study conducted in Malaysia from June to December 2020, Cheng et al. found a significant deterioration in glycemic control in 30 patients with already diagnosed T2DM [134]. An increase of HbA1c was observed (8.5 ± 0.40 pre-lockdown vs. 9.9 ± 0.46% post-lockdown). Male patients and pubertal adolescents were considered at a higher risk. Weight and BMI was surprisingly reduced in this group of patients, possibly due to the worsening of diabetes control [134]. Trieu et al. have considered the rate of new-onset T2DM during the period from April to November 2020 at a large tertiary care children’s hospital in Alabama and compared it to the rates of new-onset T2DM during the same time frame in 2019 and 2018 in the same institution [135]. A 205.3% increase in the rate of new-onset T2DM diagnosis has been observed in 2020; 290 children have been hospitalized, compared to 95 and 88 children during the same time frame in 2019 and 2018, respectively. Of the 290 children admitted with new-onset T2DM, 59.3% presented with DKA, compared to 4.2% in 2019 and 5.7% in 2018 [135].

Another study conducted in Indianapolis has compared the onset rates of newly diagnosed T2DM during the period from 1 March–14 September 2020, with those during the same time frame in the previous four years. During 2020, a total of 34 new-onset T2DM cases were registered vs. 81 in the same relative periods in 2016-2019, subdivided as follows: 16 in 2016, 14 in 2017, 23 in 2018 and 28 in 2019. Statistically significant higher levels of mean serum glucose (320 mg/dL vs. 223 mg/dL, *p* = 0.01) and HbA1c (10.8 ± 2.9% vs. 9.7 ± 2.8%, *p* = 0.07) at first presentation have been found in 2020 compared to the previous years. In addition, the 2020 cohort has a higher percentage of youth with an HbA1c ≥8.5 (79.4% vs. 61.3%, *p* = 0.08), and requiring insulin at diagnosis (79.4% vs. 59.3%, *p* = 0.05) in comparison to the 2016 to 2019 cohort. In 2020, four patients presented with DKA/HHS vs. seven in the 2016 to 2019 period (9% vs. 12%, *p* = 0.73) [136]. Similar results have been found in a study conducted at the Children’s Hospital of Los Angeles, USA [137]. In this study, the total number of newly diagnosed patients with T2DM doubled from 44 in 2018 to 82 in 2020, and the total number of DKA onsets increased from below 10% in 2018–2019 up to 20% in 2020 (*p* = 0.029). The mean age at diagnosis, sex distribution and percentage of patients with a BMI >95th percentile was not significantly different throughout the study periods, as well as the median HbA1c value at diagnosis (*p* = 0.0267) [137].

Despite the increased incidence of new-onset T2DM and DKA shown by several studies, Lee et al. did not obtain the same results in Korea [138]. Data collected from 2018 to 2020 revealed that the incidence of DKA in patients with T2DM did not significantly differ annually, as well as the total number of newly diagnosed patients with T2DM (24 in 2018, 34 in 2019 and 33 in 2020). In addition, they reported no significant changes in BMI (Standard Deviation Score- SDS) and weight (SDS) between the pre-pandemic and pandemic periods [138].

In conclusion, these studies suggest that during the COVID-19 pandemic, there has been an increase in the onset rate of T2DM and DKA, and youth with T2DM had a delayed referral to hospital in comparison to previous years. A similar severity or duration of DKA in patients with T2DM between the pre-pandemic and pandemic groups has been reported in a UK study [139]. Data extracted from the aforementioned studies are reported in Table 3.

Considering that the data from the literature are confined to only a few countries, worldwide trends are difficult to detect. Therefore, further studies with larger cohorts are necessary to improve our knowledge in this field. The currently available data also suggest that the COVID-19 vaccination is extremely helpful in this population [98]. In fact, T2DM is associated with several comorbidities and acquired immunodeficiency, making affected patients at an increased risk of COVID-19-related morbidity and mortality [140]. In addition, T2DM is mostly associated with adverse outcomes in COVID-19 patients [141], though it seems that the immune response in T2DM patients following COVID-19 vaccination is not affected by the serum glucose levels, as they show optimal antibody response [142].

## 7. Conclusions

To our knowledge, this is one of the few reviews focusing on the effect of the different types of lockdowns and of the COVID-19 pandemic on nutritional and metabolic diseases in the developmental ages available in literature to date. 

Although SARS-CoV2 infection may be seen as a negative epigenetic factor influencing the development of metabolic diseases, lockdown-related nutritional alterations may exacerbate or sometimes improve pre-existing unknown conditions. 

A limitation of this review is that there are important differences in geography, health policies and containment measures implemented by different governments to control and prevent the spread of COVID-19. 

We focused our attention on EDs, being overweight, obesity, T1DM, and T2DM. A remarkable body of research has been produced on these topics, but it is not yet exhaustive given the worldwide scale of the pandemic and the absence of data from all over the world. Despite this, as for the above mentioned conditions, data so far available seem to show a general increase in the number of diagnoses; however, a worldwide overall trend is hardly detectable. Waiting for specific treatments and effective vaccines, the main strategies adopted by governments to prevent the virus spread have been social distancing and lockdowns. Unfortunately, these strategies also resulted in the loss of regular daily routines and introduced several stressors disrupting children’s and adolescents’ lives. Clearly, these restrictions have increased the development of new healthcare approaches (e.g., telemedicine), but they have also demonstrated the fragility of the pre-existing system, which was found to be inadequate to satisfy the healthcare needs of these patients and to ensure adequate prevention for at-risk populations. On the other hand, in patients with EDs, for instance, lockdown permitted a better family participation in the patient’s daily routine, often with positive outcomes. 

We hope that future research will be conducted homogeneously, with the aim of producing more consistent and comparable data. The analysis of different trends in various countries around the world may enable the development or refinement of new or pre-existing strategies to contain the nutritional and metabolic impact of new pandemics on the developmental ages. At the same time, new technologies for medical treatment must necessarily be enhanced and expanded, so that they will make it possible to reach almost every patient, whatever the next global health challenges may be.

## Figures and Tables

**Table 1:** The type of lockdown related to the COVID-19 pandemic in different countries.

Country	Type of Lockdown	Period of Lockdown	Reference
Italy	National lockdown	1st: from 9 March 2020 to 18 May 2020 2nd: from 24 December 2020 to 06 January 2021 3rd: from 15 March 2021 to 30 April 2021	Governo Italiano [21,22]
United Kingdom	National lockdown	1st: from 23 March 2020 to June 2020 2nd: from 5 November 2021 to 2 December 2021 3rd: from 6 January 2021 to 12 April 2021	Institute for Government, UK [23]
Turkey	1st lockdown: all schools have been closed (except schools catering to students with special needs). Curfew for people over the age of 65 or with compromised immune system. On April 2020 extended to people twenty and younger 2nd lockdown: curfew on people age 65 and older and people twenty and younger 3rd lockdown: nationwide lockdown	1st: from March 2020 to June 2020 2nd: from 20 November 2020 to March 2021 3rd: from 29 April 2021 to 17 May 2021	Kanbur N. et al 2020 [24]
Israel	1st lockdown: individuals were required to stay within 100 m of their residence with the exception of using essential services 2nd lockdown: the area permitted was widened to 500 m 3rd lockdown: it was widened to 1000 m (During the full lockdowns, all regular school was conducted via online platforms)	1st: from 25 March 2020 to 3 May 2020 2nd: from 18 September 2020 to 18 October 2020 3rd: from 27 December 2020 to 11 February 2021	Shneor, E. et al 2021 [25]
Brazil	Partial lockdown closing shopping malls, restaurants, fitness centers, elementary, middle and high schools, and universities. Supermarkets and drugstores started working with restrictions concerning person-to-person distance, and public transportation started working with reduced hours.	Sao Paulo: from 24 March 2020 to 10 May 2020	Nakada LYK, et al 2020 [26]

**Table 2:** The different types of lockdown and their effects on nutritional and metabolic diseases in various countries.

Country	Type of Lockdown	Effects on Eds and Nutrition Disorders	References
ITALY	NATIONAL	Daily life restrictions, stress, and exposure to social media have been commonly responsible of the onset of eating disorders in youth.	Bozzola et al., 2022 [41]
UNITED KINGDOM	NATIONAL	Change of daily life routine and loss of self-control, worsening of symptoms, more time spent online	Dawn Branley-Bell et al., 2020 [43]
TURKEY	NATIONAL	42.1% reported feeling an improvement, 36.8% reported no change and 21.1% reported that they felt their ED was worse.	Akgul et al., 2022 [46]
ISRAEL	NATIONAL	Increase in physical activity and restricting of food eaten. Increased participation in telemedicine sessions.	Yaffa et al., 2021 [47]
BRAZIL	PARTIAL	Worsening of eating habits, especially in lower-class population with a tendency to eat junk food.	Teixeira MT et al., 2021 [42]

**Table 3:** The effect of the COVID-19 epidemic on TDM2.

Country	Pandemic Period Considered	Type of Study	Effect on TDM2	Reference
MALAYSIA	June-December 2020	Cross-sectional	Significant deterioration in glycaemic control in 30 patients with already diagnosed T2DM.Male patients and pubertal adolescents were considered at higher risk.	Cheng HP et al. [134]
USA (Alabama)	April-November 2020	retrospective	205.3% increase in the rate of new-onset T2DM diagnosis has been observed in 2020; 290 children have been hospitalized, compared to 95 children during the same time frame in 2019 and 88 children in 2018	Trieu et al. [135]
USA (Indianapolis)	March-September 2020	retrospective	During 2020 it has been registered a total of 34 new onset T2DM vs 81 in the same periods 2016-2019.Higher serum glucose and HbA1c at presentation were found in 2020.	Neyman A et al. [136]
USA (Los Angeles)	March-August 2020	retrospective single-center medical record revie	total number of newly diagnosed patients with T2DM doubled from 44 in 2018 to 82 in 2020, and the total number of DKA onset increased from below 10% in 2018–2019 up to 20% in 2020.	Chao LC et al. [137].
KOREA	2020	retrospective	the incidence of DKA in patients with T2DM did not significantly differ annually, as well as the total number of newly diagnosed patients with T2DM	Lee et al. [138]
UK	March-May 2020	Retrospective cohort study	Over representation of T2DM in COVID-positive patients with DKA than in pre-COVID or COVID-negative groups, but with similar severity	Kepegowda P et al. [139]

## Data Availability

Not applicable.
